# Testicular Synovial Sarcoma: A Case Report

**DOI:** 10.7497/j.issn.2095-3941.2012.04.010

**Published:** 2012-12

**Authors:** Mejri Nesrine, Rym Sellami, Raoudha Doghri, Hela Rifi, Henda Raies, Amel Mezlini

**Affiliations:** 1 Department of Medical Oncology, Salah Azaeiz Institute, Tunis 1006, Tunisia; 2 Department of Pathology, Salah Azaiez Institute, Tunis 1006, Tunisia

**Keywords:** synovial sarcoma, testis, chemotherapy

## Abstract

This paper reports a case of testicular synovial sarcoma with molecular genetic analysis. A 24-year-old male presented with painless scrotal mass. Ultrasonography showed a heterogeneous mass of 66 mm × 34 mm in size involving the inguinal region. Histological examination of a surgical biopsy showed a grade III monophasic growth pattern of spindle cell proliferation. Immunohistochemical analyses indicated positive staining for pancytokeratine and epithelial membrane antigen. Cytogenetic analysis showed the presence of CYT-SSX1 mutation, and CT scan showed non-specific pleural micro-nodules with a size of 7.5 mm. The patient had an extended left orchidectomy but was lost to follow-up for 1 year. A local recurrent scrotal mass of 32 mm × 25 mm, multiple inguinal lymph nodes, and increased pleural nodules, which were confirmed by histological examination, were treated with three cycles of adriamycine and ifosfamide chemotherapy, surgical resection, and radiotherapy with complete response. After 3 months, the patient developed local recurrence and pulmonary metastases that did not respond to second-line chemotherapy based on gemcitabine and paclitaxel. The patient had dyspnea at the time of this writing and chest pain, and is under third-line chemotherapy based on Deticene after 30 months of following up. This patient died on November 16, 2012 after a resperatory failure and malignant pelural effusion. Synovial sarcoma should be considered in the differential diagnosis of soft tissue tumor and it should be aggressively treated to improve prognosis. Although our patient has shown numerous factors of bad prognosis, he has had a relatively long survival time.

## Introduction

Soft tissue sarcoma (STS) accounts for 1% of all adult malignancies^[^^1^^]^. Synovial sarcoma represents 5% to 10% of all histological types of STS^[^^1^^]^. It is a mesenchymal neoplasm that appears in children and young adults and most frequently affects the extremities. Generally, it is regarded as a high-grade tumor and patients develop metastases particularly in the lungs. Optimal therapy is unknown, and the prognosis remains guarded. Unusual locations have been reported, including the mediastinum, lung, and peritoneum. We report a new case of an unusual location: the testis.

## Case Presentation

**Figure 1:**
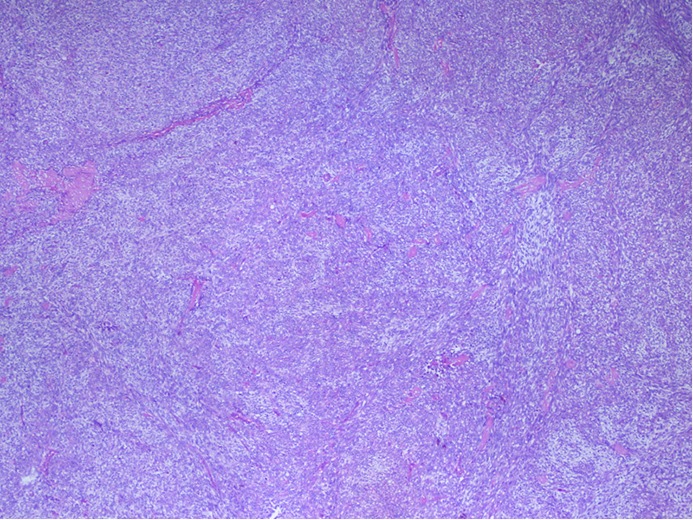
Monophasic poorly differentiated synovial saroma (H&E staining, ×20).

**Figure 2:**
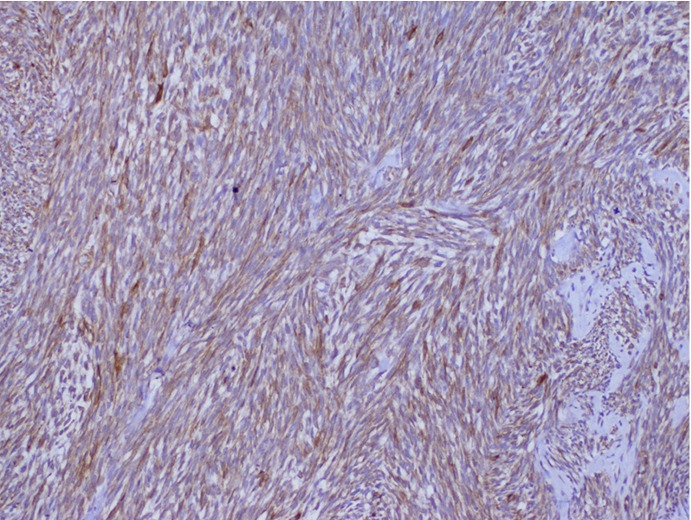
Pancytokeratine positive staining (IHC staning, ×40).

**Figure 3:**
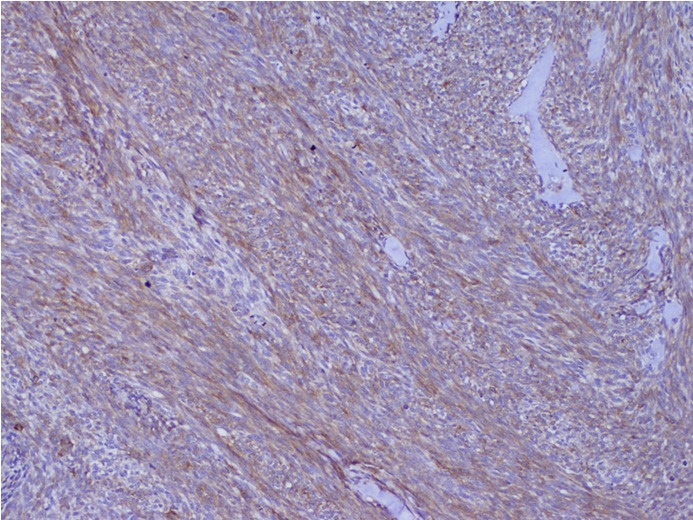
EMA positive staining (IHC staning, ×40).

In April 2010, a 24-year-old man presented with painless left testicular swelling without infection or trauma history. Clinical examination showed a firm mobile mass with a size of 62 mm × 40 mm and no palpable inguinal lymph nodes were found. He had no relevant past medical history. Scrotal ultrasonography revealed a large heterogeneous testicular mass of 66 mm × 34 mm in size in the inguinal region. Abdominal ultrasonography showed no other lesions. B human chronic gonadotrophin and α-fetoprotein levels were normal. The patient underwent surgical biopsy. Histological examination indicated a monophasic growth pattern of spindle cells (**Figure 1**). Tumor cells exhibited a hyperchromatic round or oval nucleus with features of atypia. Numerous mitoses were seen (25 mitoses/10 fields of high magnification). This proliferation infiltrated the paratesticular tunics with features of vascular emboli. Immunohistochemical analysis showed focally positive staining for pancytokeratine AE1/AE3 (**Figure 2**) and epithelial membrane antigen (EMA) (**Figure 3**). Negative results were seen with CD34, PS100, c-Kit, and desmine. Proliferative index was 60%. The tumor was classified as monophasic poorly differentiated synovial sarcoma; grade III according to the National Federation of Centers Against Cancer. A molecular biology analysis seeking t (X;18) detected the characteristic fusion transcript of SYT-SSX 1.

Computed tomography (CT) scan showed non-specific pleural micro nodules of 7.5 mm in length. Surgery with extended left orchidectomy was performed, but the patient was lost to follow-up for one year.

**Figure 4:**
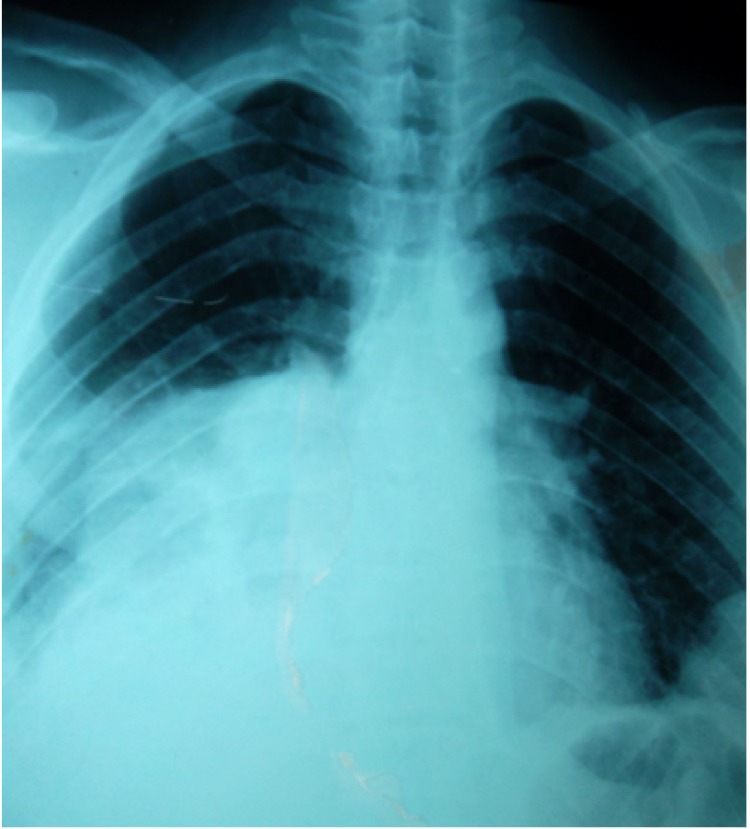
Chest X-ray showing a large pleural and pulmonary metastatic mass.

In May 2011, he presented with local recurrent scrotal mass of 32 mm × 25 mm in size and multiple inguinal lymph nodes. Chest X-ray and CT scan showed an increase in pleural nodule size and number (**Figure 4**). The recurrence was confirmed by surgical biopsy. Systemic chemotherapy based on three cycles of adriamycine and ifosfamide was performed. The evaluation showed a decrease in scrotal, inguinal, and pleural masses. The patient had surgical resection and radiotherapy at 54 Gy with complete response. Histological examination revealed the same characteristics as the first tumor. Three months after radiation therapy, a physical examination showed local recurrence. Chest X-ray showed the appearance of pulmonary metastases. Second-line chemotherapy based on three cycles of gemcitabine and paclitaxel did not inhibit tumor progression, and chest X-rays showed an increasing number of lesions. The patient was suffering from dyspnea and chest pain at the time of this writing. The total follow-up period was 30 months. The patient then underwent third-line chemotherapy based on dacarbazine (Deticene) with a stable disease. The patient died on November 16, 2012 of a resperatory failure and malignant plural effusion.

## Discussion

Synovial sarcoma originates from mesenchymal tissues. It commonly presents as an asymptomatic slow-growing tumor and predominates in adolescents and young adults between 15 and 40 years^[^^1^^]^. It occurs in extremities in 60% cases^[^^1^^]^. Testicular synovial sarcoma as primary tumor or as metastases has never been reported in the literature. We believe that this is the first case with molecular biology analysis and relatively long-term follow-up.

Histological features correspond to epithelial cells resembling those in carcinoma and spindle cells resembling those in fibrous sarcoma. Thus, synovial sarcoma is classified into four subtypes: biphasic type (presence of both epithelial and spindle cell components), monophasic epithelial type, monophasic fibrous type, and undifferentiated type. Poorly differentiated synovial sarcoma shows a high-grade appearance with high cellularity, high mitotic activity, and high atypia^[^^1^^]^. Immunohistochemical analysis indicates positive staining for EMA (**Figure 2**), cytokeratin (19, 7, AE1/3), CAM 5.2, vimentin, and Bcl2.

The differential diagnosis includes fibrous tumors of the pleura (CD34), and malignant peripheral nerve sheath tumor (PS100). Massive tumor calcification and numerous intratumoral mast cells are associated with poorer prognosis^[^^2^^]^.

Karyotyping with fluorescent in situ hybridization and genetic molecular analysis constitute the only methods to confirm the diagnosis, especially in poorly differentiated forms. Translocation (X;18) resulting in CYT-SSX gene fusion is the cytogenetic characteristic. The two mutations, CYT-SSX1 and CYT-SSX2, are correlated with the biphasic and monophasic forms, respectively. CYT-SSX1 seems to be associated with a more aggressive form of synovial sarcoma^[^^3^^]^. Tumor size (>5 cm), mitotic activity, age, and microscopic margins are the most relevant independent prognostic factors^[^^4^^]^.

Synovial sarcoma is generally treated and considered a high-grade sarcoma with frequent metastases. At an age older than 20 years at diagnosis, the size of the tumor is more than 5 cm, and the undifferentiated subtype are the main prognostic factors. Histological features such as intratumoral mast cells, tumor necrosis, high mitotic rate, and vascular invasion suggests aggressive tumor behavior.

Five-year and 10-year overall survival rates are between 24%-68% and 11%-56%, respectively^[^^1^^]^. Therapy mainly includes surgical resection with adequate safety margins. Radiotherapy showed a risk reduction of local recurrence from 7% to 50%. It is also recommended in case of positive resection margins. Chemotherapy is administered in metastatic cases, or if surgery was incomplete, it can be given in recurrence cases. A combination of ifosfamid and doxorubicin was used in the first line therapy. A combination of gemcitabine and docetaxel is another therapeutic option^[^^5^^]^. Prognosis used to be poor with a survival period of only two months, but with aggressive chemotherapy, a survival rate of 14 years has been reported^[^^1^^]^.

Although our patient presented many factors of poor prognosis, such as age, tumor size, and high mitotic activity, he has had a relatively long survival. The patient was probably already metastatic at diagnosis, but the disease seemed to have a low progressive course. Surgery, radiotherapy, and chemotherapy in case of recurrence or metastases are probably optimal treatments. This case leads us to think whether synovial sarcoma in the testis has a better prognosis.

New targeted immunotherapies are emerging. NY-ESO-1 is a cancer testis antigen expressed in normal testis tissue and in some tumors, such as breast cancer, myxoid/round cell liposarcoma, melanoma (25%), synovial sarcoma (80%)^[^^6^^]^. It is coded by a gene in the Xq28 region and is detected by RT-PCR of mRNA or by immunohistochemical staining, which can have a diagnostic value in ambiguous cases. Recent clinical trials using genetically modified T-cells that express a T-cell receptor targeting NY-ESO-1 showed the first successful case of immunotherapy in synovial sarcoma with an objective response of 45% to 67% and low toxicity because no normal tissue expresses this antigen. Furthermore, testis tissue is not targeted because it does not express a class I major histocompatibility complex. Patients should be encouraged to take part in these clinical trials^[^^7^^]^.

## Conclusion

In summary, although synovial sarcoma should be considered in the differential diagnosis of soft tissue tumor in unusual locations, it should be aggressively treated with complete resection or multimodal therapy to improve the prognosis of patients.
